# Dose-response relationship of resistance training for muscle morphology and strength in elderly cancer patients: A meta-analysis

**DOI:** 10.3389/fmed.2023.1049248

**Published:** 2023-04-06

**Authors:** Haiting Zhai, Hongwen Wei, Jixiang Xia, Wei Wang

**Affiliations:** ^1^School of Basic Sciences for Aviation, Naval Aviation University, Yantai, China; ^2^School of Strength and Conditioning Training, Beijing Sport University, Beijing, China

**Keywords:** resistance training, elderly cancer patients, upper limb strength, lower limb strength, muscle morphology, meta-analysis

## Abstract

**Objective:**

To systematically evaluate the effects of resistance training (RT) on muscle strength and muscle hypertrophy in elderly cancer patients, and to provide dose–response relationships of RT variables that could improve muscle strength and morphology in elderly cancer patients.

**Method:**

The Review Manager 5.3 was applied to analyze the 12 literatures (616 participants) through random or fixed effects model and global effect size to examine upper limb strength, lower extremity strength, and muscle hypertrophy. Sub-group analysis was made on five variables: the total number of repeated training times/week, load intensity, exercise frequency/week, exercise duration and gender. This study also examines the heterogeneity and publication bias.

**Results:**

Twelve literatures (616 participants, 60–80 years) were included in meta-analysis. RT significantly increased the upper limb muscular strength (SMD = 0.51, 95% CI: 0.10–0.93; *Z* = 2.41; *p* = 0.02) and lower extremity strength (SMD = 0.48, 95% CI: 0.28–0.67; *Z* = 4.82; *p* < 0.00001), but had no significant effect on muscle morphology(SMD = 0.21, 95% CI: 0.01–0.42; *Z* = 1.88; *p* = 0.06). In subgroup analysis for lower extremity muscle strength in elderly male cancer patients, it was found that male intensity of 70–90%1RM, volume of 400–500 times per week, frequencies of 3 times per week, and session of 12–24 weeks, revealed the greatest effect. Funnel plot of the three studies shows that the results were reliable, and no publication bias was found.

**Conclusion:**

RT had medium effects on improving muscle strength in elderly cancer patients, but it is not effective in improving muscle hypertrophy. In addition, when RT is performed, different training protocols can have an effect on the growth of muscle strength. Therefore, a lower extremity training protocol with a training intensity of 70–90% 1RM, a total of 400–500 repetitions per week, 3 times per week, and an exercise session of 12–24 weeks is most effective in improving lower extremity strength in elderly male cancer patients.

## 1. Introduction

Cancer has become one of the serious threats to human health and is predominantly seen in elderly patients, who account for approximately 50% in all newly diagnosed cancer cases, and the cancer mortality rate is as high as 71% in people aged 65 years and older (1). For most elderly cancer patients, cancer cachexia (CC) is the deadliest factor. It is mainly a secondary reaction characterized by decreased calorie intake and metabolic abnormalities caused by tumor derived factors. It is manifested in clinical syndromes such as decreased muscle strength and mass, accompanied by massive fat consumption, weight loss, etc., (2–4). Also, with the increase of the age of the elderly, the strength and quality of muscles also show a downward trend, and they often suffer from skeletal muscle reduction. Skeletal muscle reduction will continue to worsen the condition of CC patients, eventually leading to an increase in the mortality of elderly cancer patients. Therefore, there is an urgent need to explore ways to improve muscle strength and muscle morphology in older cancer patients to delay disease progression and increase the chances of survival. A review of studies has shown that resistance training (RT) in cancer patients is effective in slowing the rate of muscle atrophy and increasing muscle strength and muscle hypertrophy, but previous studies have focused on middle-aged patients or those receiving only adjuvant therapy (5). In addition, existing studies lack information on the optimal RT load training protocol for elderly cancer patients and the safety of training remains controversial (6, 7). Therefore, the main objective of this study was to evaluate the effects of RT on muscle strength and muscle morphology in elderly cancer patients through a systematic review and meta-analysis, with the aim of recommending the optimal RT regimen for elderly cancer patients.

## 2. Methods

### 2.1. Literature retrieval

**Figure 1:**
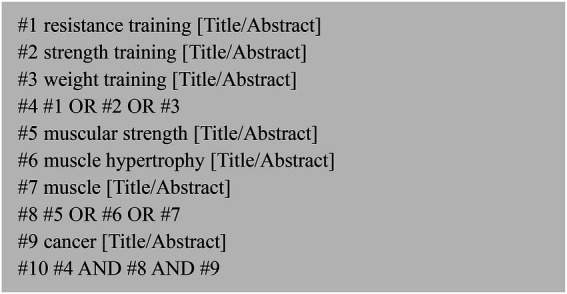
PubMed database search strategy.

The literature search was completed by researcher (Haiting Zhai) independently, mainly by searching PubMed, web of science, the Cochrane Library. The literature search was conducted from January 1st, 2000 to September 2022, with a final search date of September 20, 2022. The English search terms are: RT, strength training, weight training, muscular strength, muscle hypertrophy, muscle, cancer. The retrieval strategy takes PubMed database retrieval as an example, (# 1RT [title/abstract] or #2 strength training [title/abstract] or #3 weight training [title/abstract]) and (#4 muscular strength [title/abstract] or #5 muscle hyperplasia [title/abstract] or #6 muscle [title/abstract]) and (#7 cancer [title/abstract]) (Figure 1). The retrieved literature from each database was imported into EndNote X9 for further primary screening and inclusion according to the PRISMA statement.

### 2.2. Selection criteria

Studies with the following criteria were included in this review. (1) type of literature: paired trials or randomized controlled trials; (2) subjects were cancer patients aged 60 years or older who were actively receiving treatment or were undergoing long-term follow-up; (3) sample size, age, and sex of subjects were provided; (4) resistance experimental protocols were intervened for at least 8 weeks under the supervision of a safety organization; (5) detailed experimental design and steps were available for the study; (6) outcome indicators were muscle morphology or muscle enlargement.

The following are the exclusion criteria for the article. (1) duplicate literature; (2) subjects with additional interventions such as aerobic training or dietary supplements; (3) review and animal experiment literature; (4) non-Chinese and English literature; (5) literature without full text and otherwise unavailable; (6) literature with poor experimental design and steps; (7) literature with inconsistent outcome indicators; and (8) literature that did not meet the inclusion criteria.

### 2.3. Literature data and outcome indicators extraction

The study was conducted by two searchers using an independent double-blind approach to extract and enter relevant indicators from the literature that was eventually included in the study. The information of the entered publications included: author and time of publication, gender, sample size, age, cancer type, treatment type, and intervention protocol (load, Intensity, weekly exercise frequency, Session) (Table 1).

**Table 1:** Basic characteristics of included studies.

Author, Year	Sample size		Age		Type of cancer	Type of treatment	Intervention programs			
	RT	CON	RT	CON			Session	Times/week	Frequency	Intensity
Alberga et al. (2012)	40 (M)	41 (M)	67.1 ± 6.9	65.4 ± 7.6	Prostate cancer	Androgen blocking therapy	24	3	10 × 2 × (8–12)	60–70% 1RM
Benton et al. (2014)	8 (F)	12 (F)	68.3 ± 6.8	NA	Breast cancer	No treatment	8	2	8 × 3 × (8–12)	50–80% 1RM
Cormie et al. (2013)	10 (M)	10 (M)	73.1 ± 7.5	71.2 ± 6.9	Prostate cancer	No treatment	12	2	8 × (2–4) × (8–12)	80–100% 1RM, 6RM
Cormie et al. (2014)	20 (M17, F3)	15	70.0 ± 9.8	NA	Prostate cancer	No treatment	12	2	8 × (2–4) × (8–12)	80–100% 1RM, 6RM
Nilsen et al. (2015)	28 (M)	30 (M)	66.0 ± 6.6	66.0 ± 5.0	Breast cancer	Androgen blocking therapy	16	3	9 × (1–3) × (6–10)	8–12 RM
Nilsen et al. (2016)	12 (M)	11 (M)	67.0 ± 7.0	64.0 ± 6.0	Prostate cancer	Radiotherapy	16	3	9 × (1–3) × (6–10)	8–12 RM
Rosenberger et al. (2017)	10 (M8, F2)	10	65.0 ± 11.0	61.0 ± 6.0	Prostate cancer	Androgen blocking therapy	12	2	8 × 2 × 12	12 RM
Segal et al. (2009)	40 (M)	(M8, F2)	66.4 ± 7.6	65.3 ± 7.6	Kidney cancer	Radiotherapy	24	3	10 × 2 × (8–12)	60–70% 1RM
Simonavice et al. (2017)	27 (F)	41 (M)	64.0 ± 7.0	NA	Gastrointestinal cancer	Tyrosine kinase	24	2	10 × 2 × (8–12)	60–80% 1RM
Winters-Stone et al. (2011)	52 (F)	27 (F)	62.3 ± 6.7	62.2 ± 6.7	Prostate cancer	Inhibitor therapy	48	3	10 × (1–4) × (8–12)	60–70% 1RM
Winters-Stone et al. (2012)	36 (F)	54 (F)	62.3 ± 6.7	62.2 ± 6.7	Breast cancer	Androgen blocking therapy	48	3	9 × (1–3) × (8–12)	60–80% 1RM
Winters-Stone et al. (2015)	29 (M)	31 (F)	69.9 ± 9.3	70.5 ± 7.8	Breast cancer	No treatment	48	3	10 × 3 × (10–12)	60–70% 1RM

According to the content of the retrieved literature, upper and lower extremity maximal strength (1 Reptition Maximal, unit, kg) was used as the outcome indicator of muscle strength, lean body mass (unit: kg) was used as the primary outcome indicator of muscle hypertrophy, and muscle fiber cross-sectional area (unit: μm^2^) as a secondary outcome indicator.

### 2.4. Quality assessment of the literature

The quality of the 11 publications was assessed using the Cochrane risk of bias assessment (8). Seven items were assessed: random assignment method (A), allocation protocol concealment (B), subject and investigator blinding (C), assessor blinding (D), outcome data completeness (E), selective reporting of study results (F), and other biases (G). Each article was scored with “YES,” “NO,” and “unclear,” with “YES” being scored as 1 and “NO” or “unclear” as 0. A total score of less than 3 is considered low quality literature, 3–4 is considered medium quality literature, and 5 or more is considered high quality literature.

### 2.5. Data analysis

The analysis was performed using Review Manage 5.3, and the outcome indicators were continuous variables. Because the units of each index are different, the standard mean difference (SMD) and 95% confidence interval are used as the combined effects (9). When the SMD value is less than 0.2, it is minimal; when the SMD value is 0.2 ~ 0.5, it is small; when the SMD value is 0.5 ~ 0.8, it is medium; and when the SMD value is greater than 0.8, it is large. I^2^ is used to judge the heterogeneity between studies. If *I*^2^ < 50%, the fixed effect model is used; and if *I*^2^ ≥ 50%, the random variable model is used (2). Sensitivity analysis was performed to find sources of heterogeneity. Publication bias was tested using funnel plots.

## 3. Results

**Figure 2:**
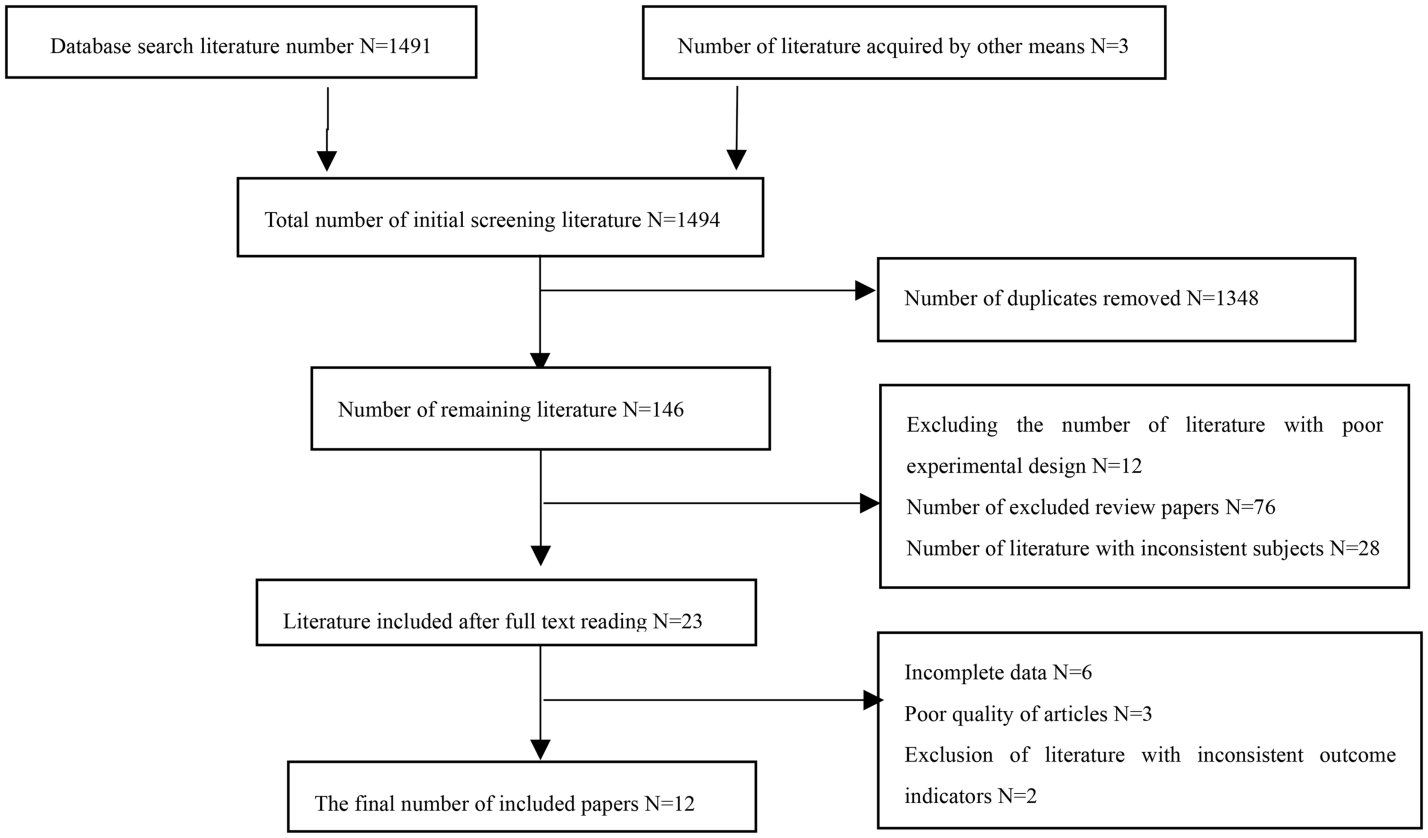
Flow diagram of study selection.

A total of 1,494 literatures were retrieved, 1,453 in English, 38 in Chinese, and 3 literatures were obtained by other means, leaving 146 literatures after deleting duplicates. According to the inclusion and exclusion criteria, 23 literatures were obtained after the initial screening of the apparently incompatible literatures, after reading the full text carefully, 12 literatures were finally included in Meta-analysis. The flow of the literature identification and selection process is outlined in Figure 2.

### 3.1. Basic information of literature inclusion

A total of 12 literatures were included in the meta-analysis (10–21). The articles were published from 2003 to 2017, and the subjects were aged 60- to 80 years, with a total sample size of 616 (experimental group: 312, control group: 304; Table 1). In 8 of the 12 studies, RT was performed during adjuvant therapy (chemotherapy, radiotherapy, androgen blockade therapy), and in 4 cases the experimental intervention was performed after completion of adjuvant therapy. The RT involved exercises such as Smith barbell squats, trainer leg stirrups, machine lifts, bench press, seated rowing, dumbbell shoulder raises, dumbbell curls, seated pulldowns, posterior neck arm curls, weighted curls, and weighted back extensions. Training volume / times (number of movements x sets x times) range: 8 × 1 × 6 ~ 10 × 4 × 12, exercise frequency/week range: 2–3 times, exercise session range: 8–48 weeks.

### 3.2. Evaluation of the quality of the literature

**Figure 3:**
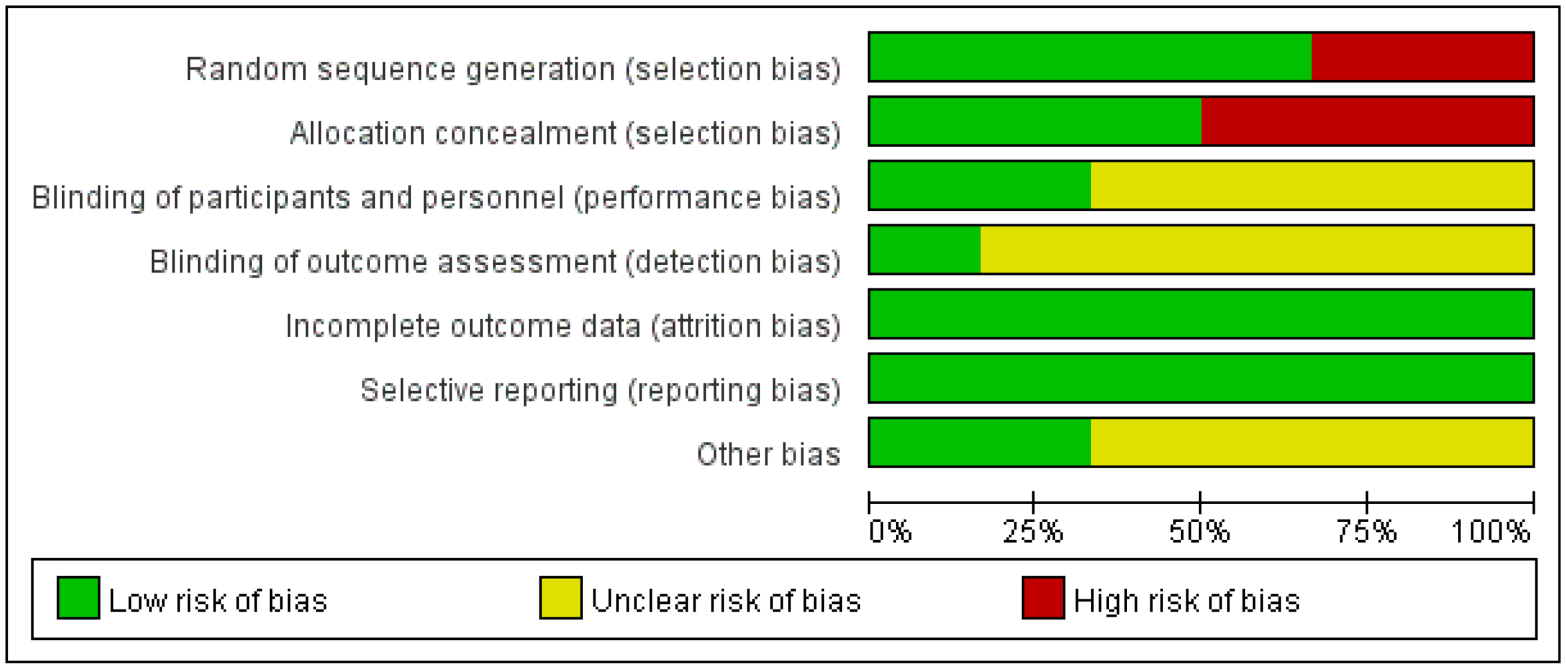
Diagram of the included literature with quality assessment. Green represents low risk, yellow represents unclear risk, red represents high risk.

**Figure 4:**
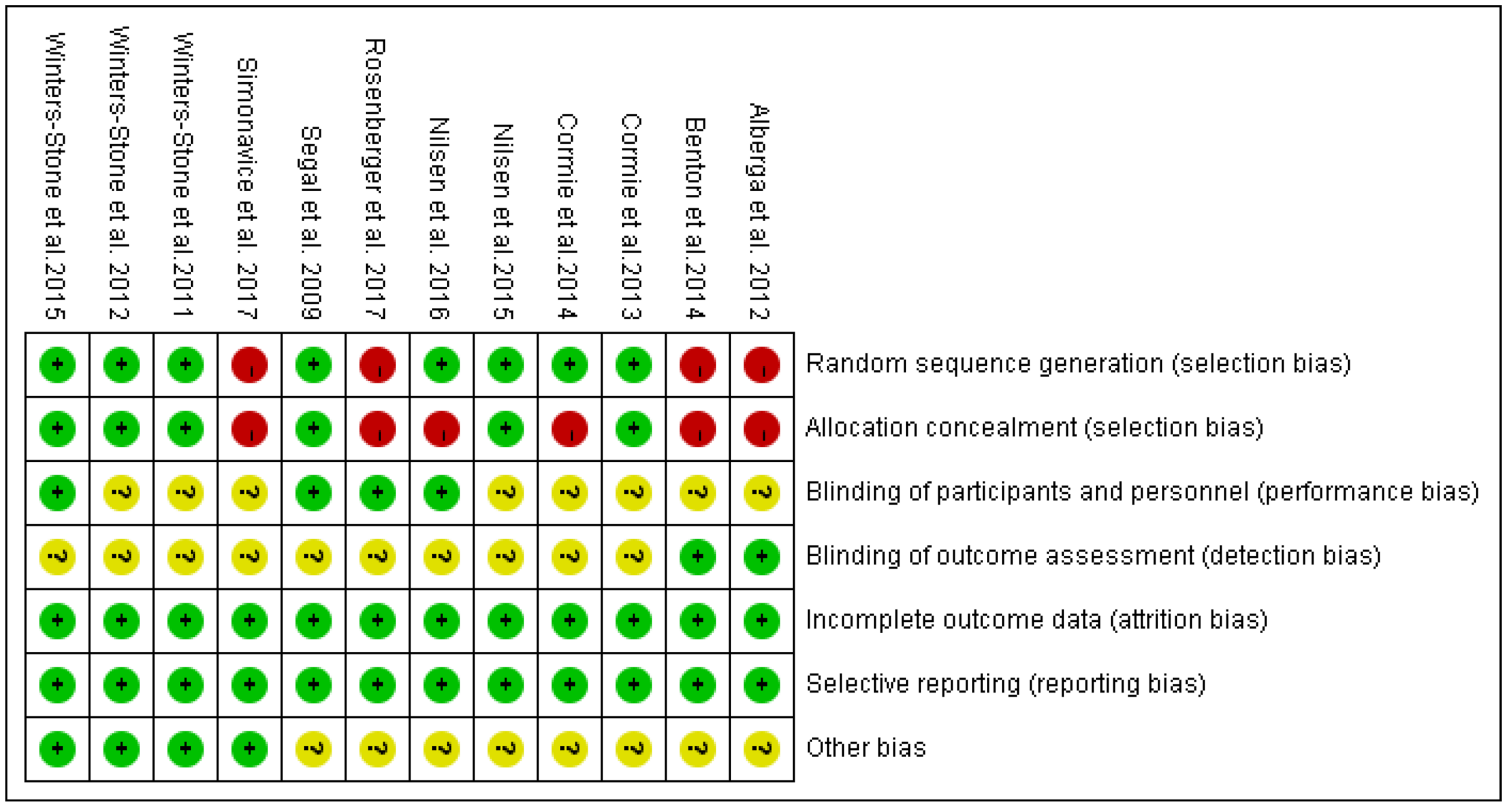
Summary diagram of the included literature with quality assessment. Green represents low risk, yellow represents unclear risk, red represents high risk.

Nine of the literatures (12–15, 17, 19–22) included in this study were randomized by number, lottery or district group (low risk), and the remaining 3 (10, 11, 16, 18) did not mention the way of random sequence generation (unclear). Four (14–17) dealt with allocation hiding, and the remaining 8 (10–13, 18–21) did not deal with allocation hiding. Seven (10, 11, 15, 16, 18, 21) mentioned assessor blinding (low risk), and the remaining 5 (12–14, 17, 19, 20) did not specify assessor blinding (unclear). Thirteen (10–21) literature all showed complete data results (low risk). None of the 12 literatures had selective reporting or other bias (low risk). Five (11, 17, 19–21) high-quality and seven (10, 12–16, 18) medium-quality literature of the included studies are shown in Table 2, and the bias results of each study are shown in Figures 3, 4.

**Table 2:** Results of subgroup analysis on upper limb muscular strength.

Subgroup analysis	Heterogeneity test			Group	Effect size and 95% confidence interval	Two-tailed test		Number of literature	Sample size
	*I*^2^ (%)		*p*			*Z*	*p*		
Gender	75	0	0.83	Male	0.46 [0.22, 0.70]	3.73	0.0002	4	270
		95	<0.0001	Female	2.94 [−2.83, 8.71]	1.00	0.32	2	83
Intensity	71	0	0.88	50–70% 1RM	0.41 [0.15, 0.67]	3.07	0.002	4	233
		94	<0.0001	70–90% 1RM	1.33 [−0.04, 2.70]	1.91	0.06	3	141
Frequency/week	71	0	<0.0001	2 Times	0.35 [−0.11, 0.81]	1.17	0.24	3	74
		46	0.68	3 Times	0.48 [0.18, 0.78]	3.58	0.0003	5	338
Number of repetitions total volume/week	71	88	0.0002	<500 Times	1.64 [−0.09, 3.38]	1.86	0.06	3	94
		0	0.66	≥500 Times	0.35 [0.12, 0.59]	2.92	0.003	4	280
Session	71	88	0.009	<24 weeks	1.64 [−0.09, 3.38]	1.86	0.06	3	94
		0	0.84	≥24 weeks	0.35 [0.12, 0.59]	2.92	0.003	4	280

### 3.3. Meta-analysis results

#### 3.3.1. Testing the effect of RT on muscle strength in elderly cancer patients

##### 3.3.1.1. Effect of RT on upper limb strength in elderly cancer patients

**Figure 5:**
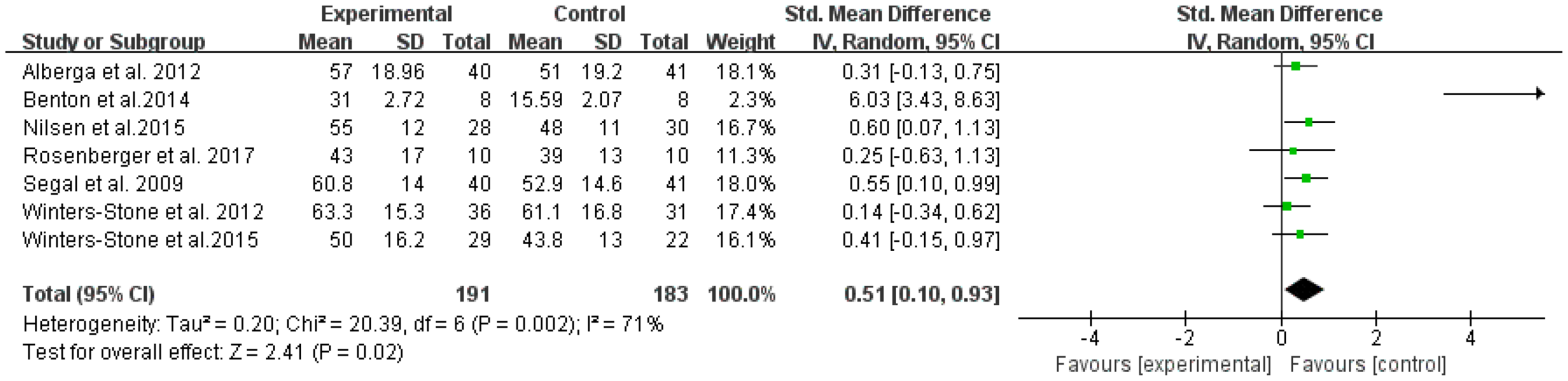
Resistance training on upper limb muscular strength in elderly cancer patients: meta-analysis.

Six literatures with outcome indicators involving upper limb strength, with a total of 374 subjects, were examined for overall effects (Figure 5). The results showed that RT was effective in improving upper limb muscle strength in elderly cancer patients with an effect size SMD = 0.51, (95% CI: 0.10–0.93; *Z* = 2.41; *p* = 0.02). Heterogeneity test of the included studies (*I*^2^ = 71% *p* = 0.01), indicated moderate heterogeneity among studies. Therefore, a random effects model was used.

##### 3.3.1.2. RT effect test on lower extremity strength in elderly cancer patients

**Figure 6:**
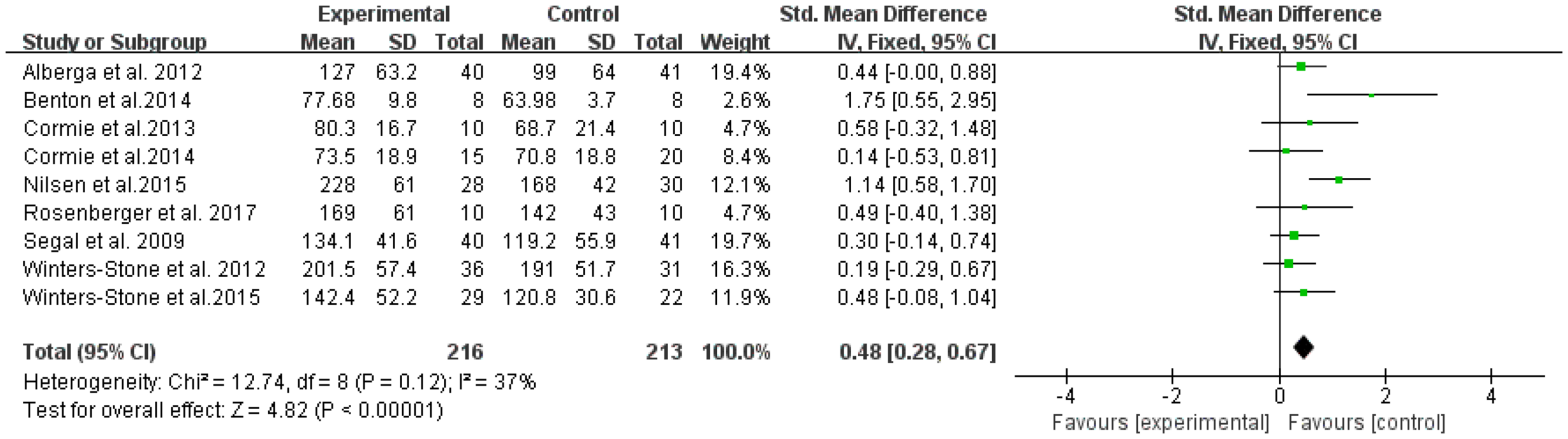
Resistance training on lower extremity strength in elderly cancer patients: meta-analysis.

The overall effect was tested on nine literatures with outcome indicators involving lower extremity strength in a total of 429 subjects (Figure 6). The results showed that lower extremity RT was effective in improving muscle strength in elderly cancer patients with an effect size of SMD = 0.48, (95% CI: 0.28–0.67; *Z* = 4.82; *p* < 0.00001). A test of heterogeneity (*I*^2^ = 37% *p* = 0.12) of the included studies indicated mild heterogeneity among studies. Therefore, a fixed effects model was used.

#### 3.3.2. RT effect test on muscle hypertrophy in elderly cancer patients

**Figure 7:**
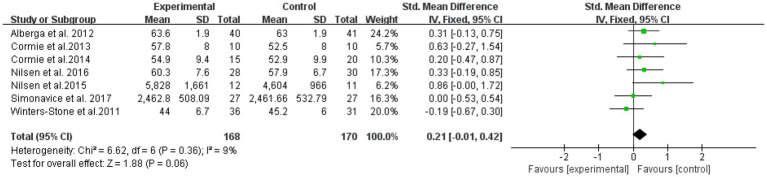
Resistance training on muscular morphology in elderly cancer patients: meta-analysis.

The overall effect was examined for nine literatures with outcome indicators involving muscle hypertrophy in a total of 338 subjects (Figure 7). The results showed that RT was not significantly effective in improving muscle hypertrophy intervention in elderly cancer patients, with an effect size SMD = 0.21, (95% CI: 0.01–0.42; *Z* = 1.88; *p* = 0.06). A heterogeneity test (*I*^2^ = 9% *p* = 0.36) of the included studies indicated mild heterogeneity between studies. Therefore, a fixed effects model was used.

#### 3.3.3. Sensitivity analysis

Sensitivity analysis was conducted separately on seven literatures included in the study of upper limb muscle strength and nine literatures included in the study of lower extremity muscle strength by transforming the effect sizes and excluding the literature one by one. It is found that there is an article (11) that leads to greater heterogeneity. By comparing with other studies, it is found that in this literature, the upper limb strength test is carried out by bench press 10 RM, and the lower extremity strength test is carried out by pedaling device. After unified conversion of 1RM, the heterogeneity of all the overall effects is *I*^2^ = 0%, *I*^2^ = 16%, respectively, which indicates that the inconsistent measurement methods of outcome indicators are the cause of moderate heterogeneity among studies. Compared with the effect quantity included in the literature, the SMD value is still in the original confidence interval, indicating that the meta-analysis results of this study have high reliability and that this study can still be retained.

#### 3.3.4. Results of subgroup analysis

Subgroup analyses of upper and lower extremity muscle strength were performed based on the review information, comparing patient gender (male or female), RT intensity (50–70% or 70–90%), frequency (2 or 3 reps/week), exercise volume (<500 and ≥500 reps/week), and session (<24 and ≥24 weeks).

##### 3.3.4.1. Subgroup analysis of RT on upper limb muscle strength in elderly cancer patients

The results of the subgroup analysis (Table 2) showed that, (1) In a subgroup analysis of the seven included papers with a total of 353 subjects, RT was effective in improving upper extremity muscle strength in male elderly patients compared to female patients. The effect size was statistically significant in the male group (*p* = 0.0002), while it was not statistically significant in the female group (*p* = 0.32). Moderate heterogeneity existed between the male and female groups (*I*^2^ = 75%). (2) Intensity of RT (7 literatures, 374 subjects in total) of 50–70% was effective in improving upper limb muscle strength in elderly patients, while intensity of 70–90% was not. There was a significant difference in the amount of effect between the 50–70% and 70–90% groups (*p* = 0.19). Intensity was statistically significant in the 50–70% group (*p* = 0.002), while the effect size was not statistically significant in the 70–90% group (*p* = 0.06), and there was moderate heterogeneity between the 50–70% and 70–90% groups (*I*^2^ = 71%). (3) Sub-group analysis of frequency/week (7 literatures, 412 subjects in total) showed that three times a week was effective in improving upper limb muscle strength of elderly patients, but twice a week was ineffective. The effect of training three times a week was statistically significant (*p* = 0.0003), but the effect of training twice a week was not statistically significant (*p* = 0.24). There was a significant difference in the amount of effect between two training groups and three training groups (*p* = 0.002). There was moderate heterogeneity in training twice and three times a week (*I*^2^ = 71%). (4) The results of subgroup analysis of exercise amount/week (7 literatures, 374 subjects in total) showed that the exercise amount/week of RT ≥500 times was effective for the upper limb muscle strength of elderly patients, but the exercise amount/week <500 times was ineffective. The effective amount of exercise/week ≥500 times was statistically significant (*p* = 0.0003), but the effective amount of exercise/week <500 times was not statistically significant (*p* = 0.06). There were significant differences between the two groups (*p* = 0.002). There was moderate heterogeneity (*I*^2^ = 71%) between the groups with less than 500 exercises/week and ≥500 exercises. (5) Session: 7 included literatures with a total of 374 subjects. The results showed that RT of session ≥24 weeks was effective for upper limb muscle strength in elderly patients, while session <24 weeks was not. The effect size was statistically significant in the ≥24 weeks session group (*p* = 0.0003), whereas it was not statistically significant in the <24 weeks session group (*p* = 0.06), and there was a significant difference in the effect size between the <24 weeks and ≥ 24 weeks session groups (*p* = 0.002). Moderate heterogeneity existed between groups with session <24 weeks and ≥ 24 weeks (*I*^2^ = 71%).

##### 3.3.4.2. RT on lower extremity muscle strength subgroup analysis in elderly cancer patients

The results of the lower extremity muscle strength subgroup analysis (Table 3) showed that (1) gender: nine included literatures with a total of 625 subjects were analyzed in subgroups. The results showed that RT was effective in improving lower extremity muscle strength in male elderly patients, but not in female patients. There was near moderate heterogeneity between the male and female groups (*I*^2^ = 49%). The effect size was statistically significant in the male group (*p* < 0.01), while it was not statistically significant in the female group (*p* = 0.10), and there was a significant difference between the male and female groups (*p* = 0.0003). (2) Intensity: subgroup analysis was performed on 591 subjects from 8 included literatures. The results showed that the two load intensities were effective in improving the lower of elderly cancer patients, and there was a significant difference between the two groups (*p* < 0.0001). The effect amount of 70–90%1RM group was the most obvious, with SMD = 0.62 (95% CI: 0.14–1.11; Z = 2.74; *p* = 0.006). There is low heterogeneity between 50 and 70%1RM group and 70–90%1RM group (*I*^2^ = 37%). The within-group effect of the two groups was statistically significant (*p* < 0.05). (3) Frequency/week: a subgroup analysis was performed on 8 included literatures with a total of 413 subjects. The results showed that RT performed 3 times per week was effective in improving lower extremity muscle strength in elderly patients, whereas it was not effective when performed twice per week. The within-group effect size was not statistically significant for training frequency of 2 sessions/week (*p* = 0.14), and the within-group effect size was statistically significant for training frequency of 3 sessions/week (*p* = 0.02), with a significant difference between groups (*p* < 0.0001). There was low heterogeneity between groups with training frequency at 2 sessions/week and 3 groups/week (*I*^2^ = 16%). (4) Exercise volume/week: subgroup analysis was performed on the 9 included literatures with a total of 439 subjects. The results showed that exercise volume/week <500 times and exercise volume/week ≥500 times were effective in improving the lower extremity strength of elderly cancer patients, and the effect volume of exercise volume/week <500 times was the most obvious, with SMD = 0.76 (95% CI: 0.25 ~ 1.27; *z* = 2.90; *p* = 0.004). The effect amount within the two groups was statistically significant (*p* = 0.004), and there was a significant difference between the two groups (*p* < 0.0001). There was low heterogeneity (*I*^2^ = 37%) between the groups with exercise volume/week <500 times and exercise volume/week ≥500 times. (5) Session: a subgroup analysis was performed on 439 subjects from 9 included literature. The results showed that the two session were effective in improving the lower extremity strength of elderly cancer patients. The effect of the session was the most obvious in the group with session shorter than 24 weeks, with SMD = 0.76 (95% CI: 0.25 ~ 1.27; *z* = 2.90; *p* = 0.004). The effect sizes within the two groups was statistically significant (*p* = 0.004), and there was a significant difference between the two groups (*p* < 0.0001). There was low heterogeneity (*I*^2^ = 37%) between groups with session shorter than 24 weeks and duration was at least 25 weeks.

**Table 3:** Results of subgroup analysis on lower extremity muscular strength.

Subgroup analysis	Heterogeneity test			Group	Effect size and 95% confidence interval	Two-tailed test		Number of literature	Sample size
	*I*^2^ (%)		*p*			*Z*	*p*		
Gender	49	0	0.24	Male	0.65 [0.31, 0.99]	3.72	0.0002	4	210
		95	0.06	Female	0.50 [−0.10, 1.10]	1.65	0.10	3	164
Intensity	37	0	0.18	50–70% 1RM	0.51 [0.14, 0.87]	2.74	0.006	4	229
		94	0.15	70–90% 1RM	0.62 [0.14, 1.11]	2.51	0.01	4	362
Frequency/week	16	0	0.70	2 Times	0.35 [−0.11, 0.81]	1.48	0.14	3	75
		46	0.72	3 Times	0.48 [0.18, 0.78]	3.17	0.002	5	338
Number of repetitions total volume/week	37	88	0.09	<500 Times	0.76 [0.25, 1.27]	2.90	0.004	5	159
		0	0.12	≥500 Times	0.34 [0.11, 0.58]	3.85	0.004	4	280
Session	37	88	0.009	<24 weeks	0.76 [0.25, 1.27]	2.90	0.004	5	159
		0	0.84	≥24 weeks	0.34 [0.11, 0.58]	2.85	0.004	4	280

#### 3.3.5. Publication bias test

**Figure 8:**
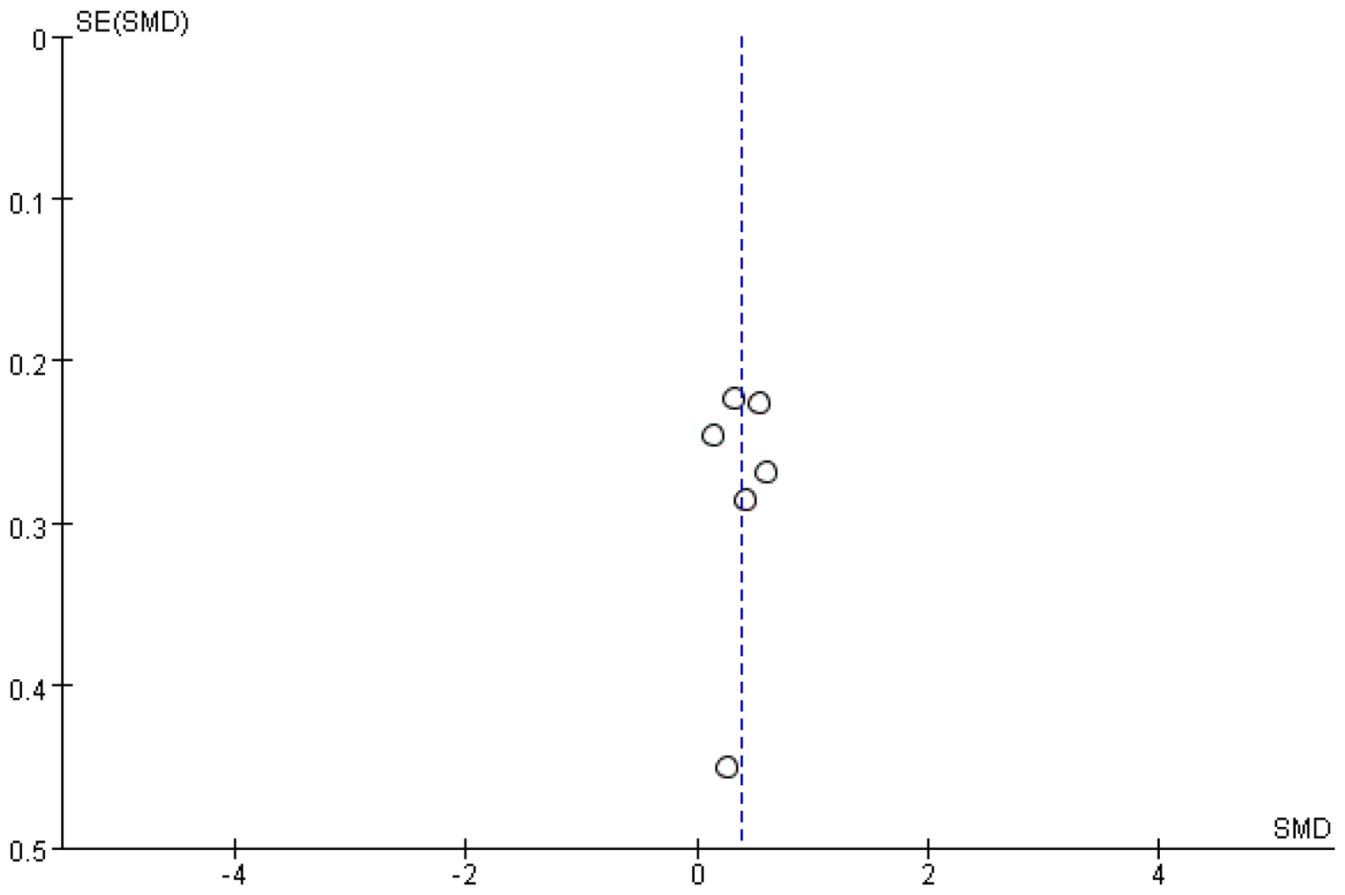
Funnel plots of upper limb.

**Figure 9:**
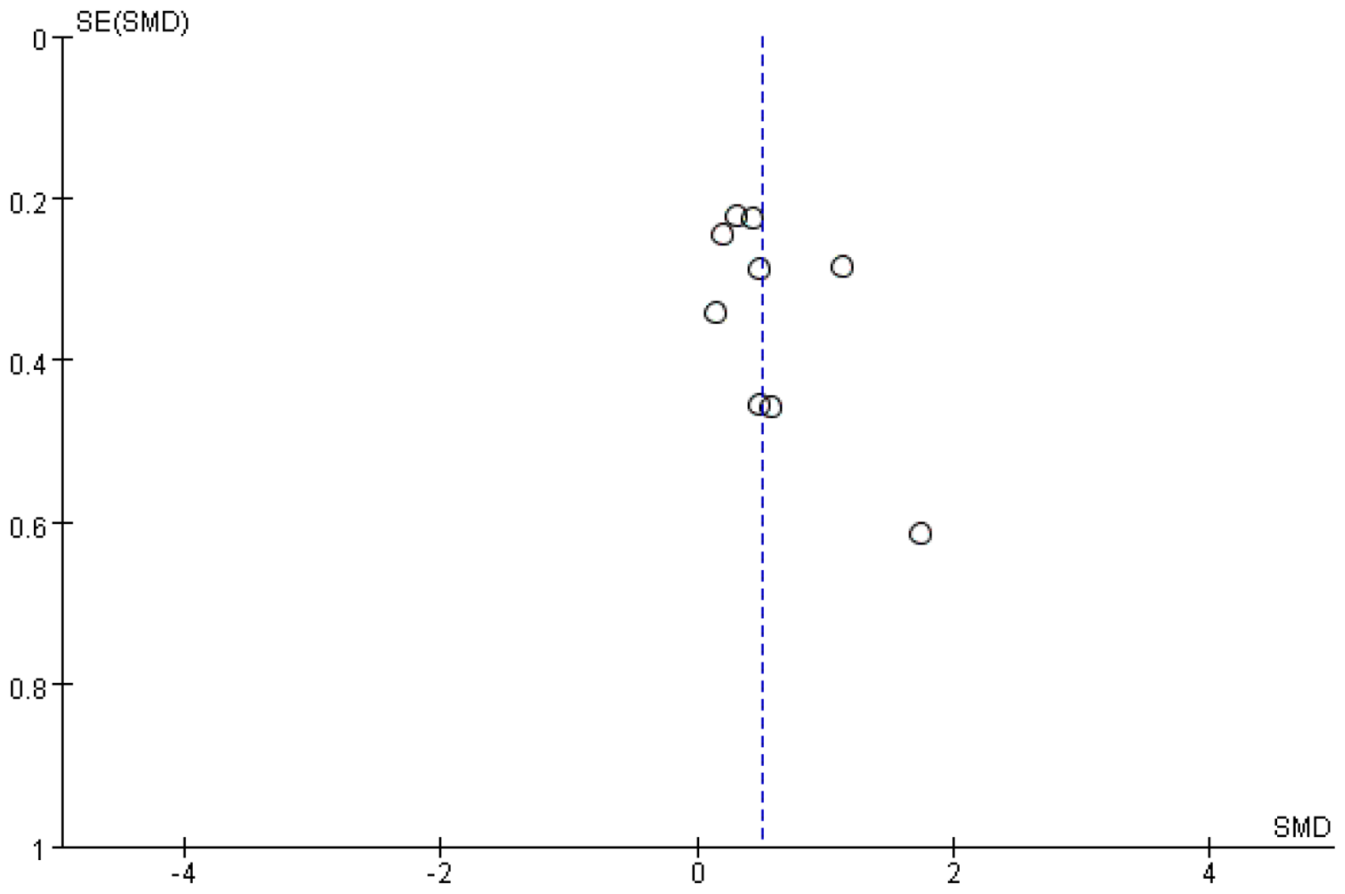
Funnel plots of upper limb.

**Figure 10:**
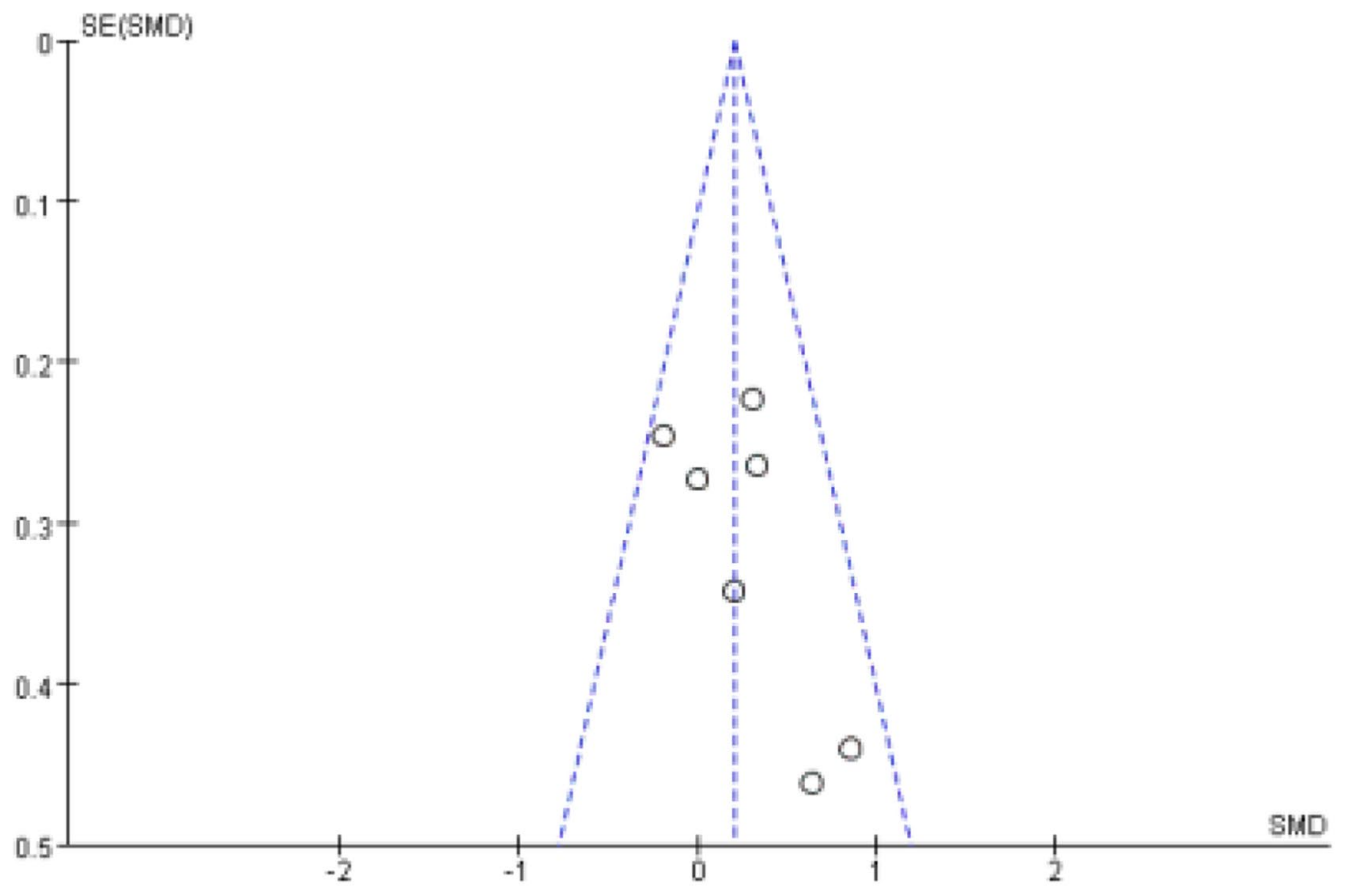
Funnel plots of muscular morphology.

Meta-analysis funnel plots of the effects of interventions on upper limb muscle strength, lower extremity muscle strength and muscle morphology in elderly cancer patients by RT showed that most of the scatter distributions were on the bias and symmetrical to each other, indicating that there was no significant publication bias among the studies and the findings were reliable (Figures 8–10).

## 4. Discussion

The purpose of this systematic review and Meta-analysis was to investigate the intervention effects of RT on muscle strength and muscle morphology in elderly cancer patients. The results of the study showed that RT could effectively improve muscle strength of upper and lower extremities in elderly cancer patients, but had no significant effect on muscle morphology improvement. The present study further validated the previous findings that RT had a moderate effect size on muscle strength improvement. However, the effect of the intervention on muscle morphology differed from the results of Barbara’s study, and the present study did not find a significant effect of RT on muscle morphology in elderly cancer patients (5). First, after the age of 50, the human body loses an average of 0.4 kg of lean weight every year (22). Although about half of cancer patients will show weight loss, in cachexia, the weight loss is mainly the significant reduction of skeletal muscle. In cachexia patients, 25% weight loss means the loss of about 75% skeletal muscle (23). Second, the response of RT to muscle hypertrophy may decrease with increasing physiological adaptation to training time, and the load of RT should be increased gradually to produce a significant effect on muscle morphology. Third, other elements of training should be moderately adjusted throughout the intervention process to ensure that muscle morphology has a significant effect. For example, the type of movement, the volume of training, and the change in strength and speed. In addition, insignificant muscle morphology may be associated with additional dietary interventions. The subjects in this study did not consume, for example, protein supplements or foods high in protein, which could also lead to a reduction in the rate of muscle protein synthesis. A review study by Germak showed that muscle hypertrophy and strength were significantly higher in elderly people using protein supplements than in non-users (24). This suggests that RT only has limited effect on improving muscle hypertrophy in elderly cancer patients over 60 years of age and that other adjuncts, such as the intake of nutritional supplements and protein products, are needed. However, there are relatively few studies on the effects of RT with protein intake on muscle hypertrophy in elderly cancer patients. Therefore, further studies are also needed in the future to determine the safety and effects of the effects.

In this study, by performing RT on cancer patients with a mean age of 66.2 years, the results showed that it could significantly increase upper limb strength by 12.6% and lower extremity strength by 19.7%. It has been suggested that a 10.7 kg increase in leg strength in men can reduce the risk rate of cancer death by 35% (24). The main mechanism is that RT increases motor neuron recruitment and excitability-induced neuromuscular adaptation, which promotes muscle cell activation during muscle contraction and reduces pro-inflammatory cytokine activity (25, 26). RT reduces cancer-related muscle atrophy by downregulating ATP-dependent ubiquitin proteasome system. The main system responsible for muscle protein degradation in cancer. In addition, the treatment process of cancer produces different degrees of side effects on the body, which usually include: muscle atrophy, decreased physical function, changes in body composition, depression, and fatigue (27). The decrease in physical activity is associated with other side effects (e.g., decreased appetite) and may further exacerbate muscle atrophy, leading to a decrease in overall muscle strength levels and increased feelings of fatigue (28, 29). The loss of muscle strength and the reduction of aerobic energy supply capacity restrict the daily activities of cancer patients, which seriously affects the quality of life of cancer patients (30). In addition, muscle loss rates above 5% are associated with 10% cancer mortality (31) and contribute to increased mortality (32–34). The results of this study showed an average increase in lower extremity strength of 25.61 kg in elderly cancer patients through RT training. Therefore, early intervention of RT can enhance muscle strength to improve the health status of elderly cancer patients.

The results showed that RT produced moderate effect sizes on both upper and lower extremity strength in older cancer patients. Subgroup analysis was used to explore sources of heterogeneity and to determine RT protocols, as RT did not have a significant effect on muscle hypertrophy in older cancer patients. Subgroup analyses were not performed in this study. When subgroup analysis was performed for gender, two of the included literatures on upper extremity strength mentioned women and four mentioned men. The literatures on lower extremity strength included 3 references to females and 4 references to males. Through the analysis of the adjusting variable of gender: in terms of upper and lower extremity strength, the differences between the two groups are statistically significant, but the research on gender is mainly focused on men; and resistance has no statistical significance on the upper and lower extremity strength of elderly female cancer patients. Therefore, the results of gender as a moderating variable need to be interpreted with caution.

The intensity of exercise may be a regulating variable that affects the effect of RT on the intervention of lower extremity strength of elderly cancer patients. The variable shows that the intervention effect of high intensity (90% 1RM) is better than that of medium intensity (75%1 RM). This is consistent with the research results of Ruiz (35). The increase of intensity enhances the physiological adaptation of muscles. First, it allows an increase in neurological involvement and an increase in the number of synchronized muscle fibers involved, which leads to an increase in the number of motor units. While the phosphorylation effect of light chain regulated by myosin is enhanced, and a series of chemical reactions lead to changes in the structure of myosin and an increase in the number of transverse bridges, thereby increasing muscle contraction strength (36). However, this is inconsistent with the findings of Nicholas, who found in his study that low loading (30% 1RM) was more effective in increasing muscle protein synthesis than high loading (90% 1RM) for lower extremity strength (37). Similarly, results from Barbara’s meta-analysis of RT in cancer survivors showed that moderate Intensity (75% 1RM) produced the largest effect sizes, which is explained by the fact that RT-induced muscle protein synthesis is not necessarily intensity-dependent, but may be determined by exercise volume (5). The subjects of the two studies were healthy adults and cancer patients aged less than 50 years, and the subjects of the present study were mainly elderly people aged 60 years or older. Therefore, age is likely to be responsible for the greater amount of lower extremity strength effect of high-intensity exercise load in the results of the present study. Older adults experience a more pronounced decline in muscle strength with age than people in other stages of life, and changes in strength require greater physiological adaptation of muscle fibers, as intensity is a key factor affecting muscle strength (38–40). Intensity showed statistically significant differences between groups in subgroup analysis of RT on upper extremity strength modifying variables in elderly cancer patients, but high intensity was not significant in improving upper extremity strength. Firstly, the included studies were too homogeneous in terms of upper extremity strength training and less than three movements. Furthermore, the amount of literature studied was not sufficient to demonstrate the effect of high-Intensity degrees of upper extremity strength training. Therefore, more studies on different load intensities are needed in the future to explore the effects on upper extremity strength in cancer patients.

The study showed that exercise volume/week may be a moderating variable affecting the effect of RT on lower extremity strength intervention in elderly cancer patients. Exercise volume/week in the range of 400–500 repetitions had a moderate effect size, and exercise volume in the range of 400–500 repetitions/week had a better intervention effect on lower extremity strength in elderly cancer patients than exercise volume ≥500 repetitions/week. Load includes two variables: the amount and the intensity. Load includes two variables: load amount and load intensity. In this study, it is found that the effect of high-intensity load on lower extremity strength is greater than that of medium intensity. Obviously, the effect of relatively low load (400–500 times/week) is greater than that of large load (more than 500 times/week). It also verifies the inverse relationship between load intensity and load. The results of Barbara’s meta-analysis of RT in cancer survivors are consistent, and the muscle strength of elderly cancer patients can be improved under the condition of medium to high training volume (5). Exercise volume/week showed statistically significant differences between groups in a subgroup analysis of RT on upper extremity strength modifying variables in older cancer patients, but the improvement in upper extremity strength with high loading volume was not significant. This may be related to other underlying factors of training. Single-session training time, rest time, and inter-set intervals were not reported in most of the included studies. Therefore, more studies are needed to validate these variables.

The results showed that frequency/week showed a statistical difference between groups in the subgroup analysis of RT on upper and lower extremity strength modifying variables in elderly cancer patients, but the frequency of 2 training times/week did not show a statistical difference in the improvement of upper and lower extremity strength, respectively, indicating that low frequency (2 times/week) did not lead to changes in muscle strength. However, there was a significant difference between 3 times/week training frequency to improve upper and lower extremity strength. Leidy’s study showed that RT performed 2 times/week had a greater amount of muscle strength, pain, aerobic capacity and quality of life improvement effects in cancer patients (41). Yet Lopez showed that resistance group training performed either 2 or 3 times a week promoted muscle strength improvement in cancer patients (42). The reason for the inconsistent conclusions may be related to the age, type of cancer, and BMI of the subjects. In addition, in terms of intervention measures, the former two review studies included more mixed training of resistance and aerobic. Most of the training guidelines in the literature included in this study took into account everyone’s physical condition. According to the recommendations of the American College of sports medicine (ACSM) to cancer survivors, sports injuries or adverse events in elderly patients should be minimized to achieve greater training effect (43).

This study have shown that session may be a moderating variable affecting the effect of RT on lower extremity strength interventions in elderly cancer patients, with exercise sessions of 8–24 weeks approaching large effect sizes and exercise sessions of 12–24 weeks outperforming interventions of ≥24 session on upper and lower extremity strength in elderly cancer patients. The results of this paper are somewhat consistent with previous intervention effects of RT session on muscle strength in older adults (43, 44). However, the results differ from those of a 24-week training period. This may be due to the lower threshold of muscle adaptation in untrained older adults during the initial phase of training and the need for greater stimulation after this initial phase, unlike what has been observed in healthy older adults (45). In addition, chemotherapy decreases bone mineral density in elderly cancer patients and the body turns out to be weak (46–48). As chemotherapy is also associated with muscle reduction, the session of RT may also be related to the fact that RT experience has an impact on response during or after chemotherapy. Therefore, in the future, the identification of those various factors associated with RT should be sought in order to establish further optimized exercise guidelines.

## 5. Study limitations and future perspectives

For this systematic review and Meta-analysis, some studies were excluded in order to enhance the homogeneity of the studies, resulting in an insufficient number of studies included in the literature. In addition, foreign language was only included in English literature, which may increase risk bias to some extent. The predominance of patients with breast and/or prostate cancer among the included cancer types limits the generalizability of this review to patients with all cancers. Although the standard error differences between randomized and non-randomized controlled trials were not significant (49, 50), not all of the included studies involved randomized sequences and blinding. Interval time, movement speed, and recovery supplementation may also be moderating variables, but were mentioned in only two studies (13, 16). Therefore, this study was not conducted to explore its effects by combining data.

The RT guidelines in this review should be applied to patients with different cancer types, considering the physical status of older adults with cancer. Future studies should go beyond the traditional ACSM guidelines and explore the effects of various training elements in order to establish further optimized exercise guidelines. For example, low-intensity RT should be performed with short rest periods between sets to achieve the recovery of muscle fatigue; high-intensity should be limited not only to the size of the weight, but also be defined as light weights with rapid repetitions and short intervals.

## 6. Conclusion

In conclusion, the systematic review and Meta-analysis showed that RT had medium effects on improving muscle strength in elderly cancer patients, but it is not effective in improving muscle hypertrophy. In addition, when RT is performed, different training protocols can have an effect on the growth of muscle strength. Therefore, a lower extremity training protocol with a training intensity of 70–90% 1RM, a total of 400–500 repetitions per week, 3 times per week, and an exercise session of 12–24 weeks is most effective in improving lower extremity strength in elderly male cancer patients.

## Data availability statement

The original contributions presented in the study are included in the article/supplementary material, further inquiries can be directed to the corresponding authors.

## Author contributions

HZ and HW: conceptualization. HZ, HW, JX, and WW: data curation and writing-original draft, writing-review, and editing. HZ, HW, and JX: formal analysis and investigation. All authors contributed to the article and approved the submitted version.

## Conflict of interest

The authors declare that the research was conducted in the absence of any commercial or financial relationships that could be construed as a potential conflict of interest.

## Publisher’s note

All claims expressed in this article are solely those of the authors and do not necessarily represent those of their affiliated organizations, or those of the publisher, the editors and the reviewers. Any product that may be evaluated in this article, or claim that may be made by its manufacturer, is not guaranteed or endorsed by the publisher.
